# Giant Calcified Pericardial Cyst With Caseous Degeneration Causing Right Ventricular Compression

**DOI:** 10.1093/icvts/ivag097

**Published:** 2026-04-09

**Authors:** Yasuhiko Kawaguchi, Badr Arbaein, Raffael Pereira Cezar Zamper, A Dave Nagpal

**Affiliations:** Division of Cardiac Surgery, London Health Science Centre, Western University, London, Ontario, N5X 5A5, Canada; Division of Anesthesiology, London Health Science Centre, Western University, London, Ontario, N5X 5A5, Canada; Division of Anesthesiology, London Health Science Centre, Western University, London, Ontario, N5X 5A5, Canada; Division of Cardiac Surgery, London Health Science Centre, Western University, London, Ontario, N5X 5A5, Canada; Critical Care Western, London Health Science Centre, Western University, London, Ontario, N5X 5A5, Canada

**Keywords:** pericardial cyst, calcification, caseous degeneration, compression, pericardiectomy

## Abstract

Calcified pericardial cysts are exceedingly rare, and cysts containing caseous material are scarcely reported. We describe a giant calcified pericardial cyst causing right ventricular compression and heart failure symptoms. Imaging demonstrated a large calcified pericardial mass abutting the right ventricle and atrioventricular groove. Surgical resection via median sternotomy with anterior pericardiectomy was performed. Intraoperatively, thick caseous material was found within a heavily calcified cyst wall. Histopathology revealed a fibrous cyst wall without epithelial lining and no evidence of malignancy or granulomatous inflammation. Post-resection transoesophageal echocardiography confirmed relief of right ventricular compression with preserved function. This case highlights diagnostic challenges and surgical considerations of complex calcified pericardial cysts with atypical intracystic contents.

## INTRODUCTION

Pericardial cysts are rare benign mediastinal lesions with an estimated incidence of 1 per 100 000 individuals.^1^ They are typically thin-walled, non-calcified, and asymptomatic. Calcification of a pericardial cyst is distinctly uncommon and may complicate diagnosis and surgical management.^2^ Furthermore, the presence of caseous intracystic material broadens the differential diagnosis to include chronic inflammatory processes, prior haemorrhage, or mycobacterial infection.^3^ We report a rare case of a giant calcified pericardial cyst with caseous degeneration producing clinically significant right ventricular (RV) compression.

## CASE REPORT

A 66-year-old man with a remote history of blunt chest trauma from a motor vehicle accident presented with progressive dyspnoea, atypical chest discomfort, and peripheral oedema. His medical history included hypertension and dyslipidaemia. Transthoracic echocardiography (TTE) demonstrated a homogeneous extracardiac mass compressing the RV cavity, with borderline RV systolic function and no intracavitary gradient (**Video S1**). Left ventricular systolic function was low-normal.

**Figure 1.**
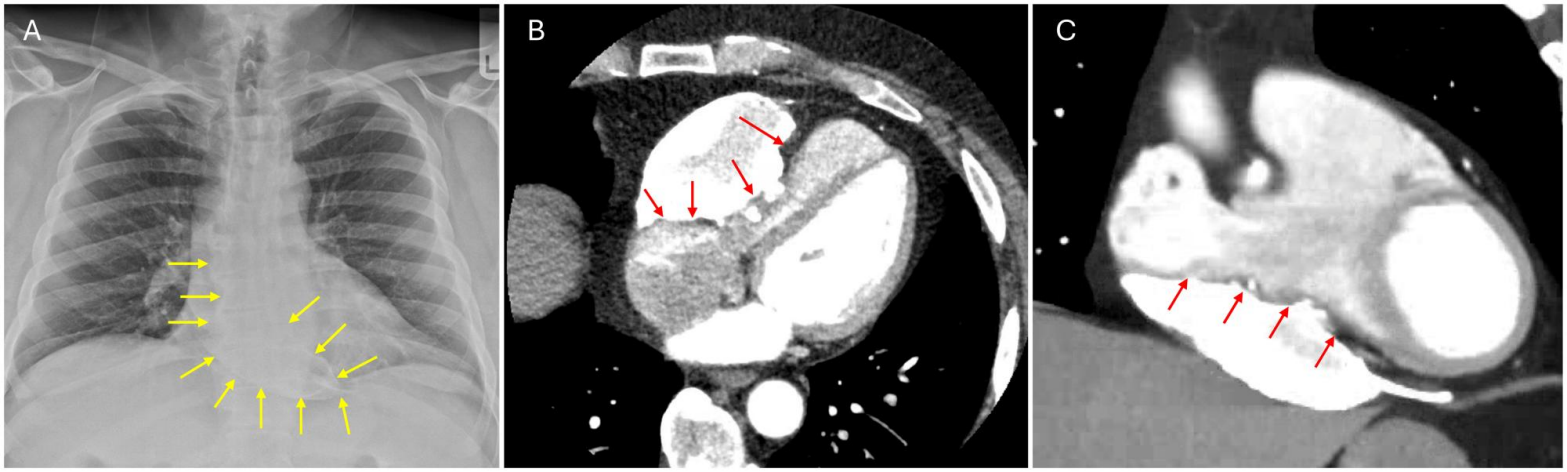
Preoperative Imaging. (A) Chest radiograph showing pericardial calcification (arrow). (B) Axial and (C) coronal computed tomography angiography demonstrating a calcified pericardial mass (arrows).

Computed tomography angiography revealed a well-defined, heavily calcified pericardial mass along the RV free wall, extending to the base of the heart and atrioventricular groove, with focal calcification involving the RV wall. The mass measured approximately 5 × 11 cm and caused compression of the RV and right atrial appendage (**Figure 1**, **Video S2**). Retrospective review of a chest radiograph obtained over 10 years earlier demonstrated pericardial calcification, suggesting a chronic process (**Figure 1A**). Coronary angiography showed normal coronary arteries without external compression. Differential diagnoses included a calcified pericardial cyst, chronic organized haematoma, pericardial tumour, and sequelae of prior pericarditis. Surgical exploration was undertaken to relieve mechanical cardiac compression.

**Figure 2.**
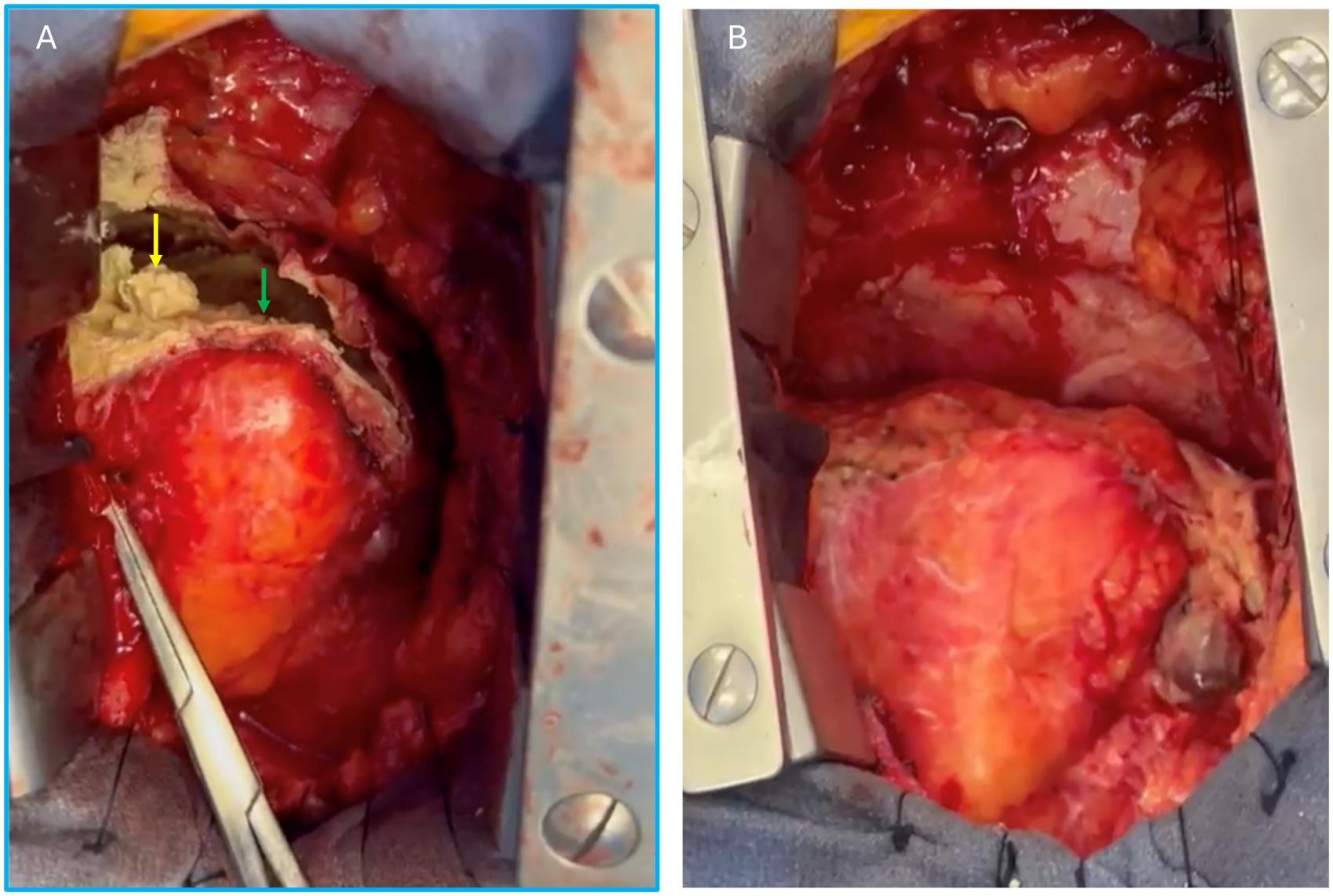
Intraoperative Findings. (A) Calcified cyst wall (green arrow) with caseous material (yellow arrow). (B) Operative field after cyst resection.

Median sternotomy revealed dense pericardial adhesions and a large, rigid calcified mass overlying the right ventricle. Upon opening the cyst, thick white caseous material was expressed and sent for microbiological and pathological analysis (**Figure 2A**). Dissection confirmed a predominantly pericardial origin. The cyst was excised as completely as safely possible (**Figure 2B**); however, densely adherent calcification at the atrioventricular groove was left in situ to avoid myocardial injury (**Video S3**). An anterior pericardiectomy from phrenic nerve to phrenic nerve was performed. Intraoperative transoesophageal echocardiography (TEE) demonstrated complete relief of RV compression with preserved function (**Video S4**). Cardiopulmonary bypass was not required.

Histopathology showed a fibrous cyst wall devoid of epithelial lining, consistent with a pericardial cyst, without malignancy or granulomatous inflammation. Microbiological cultures, including mycobacterial studies, were negative. The postoperative course was uneventful, and the patient was discharged on postoperative day 3. He remained asymptomatic at 6 month, and follow-up echocardiography showed normal biventricular function without compression or RV diastolic dysfunction.

## DISCUSSION

Calcified pericardial cysts are exceptionally rare and may mimic other pericardial or cardiac pathologies, including teratomas, chronic haematomas, or constrictive pericardial disease.^1^^,^^3^ The presence of caseous intracystic material further complicates diagnosis. Teratoma may contain tissues such as hair, teeth, or bone which may appear as high-density components on CT or recognized intraoperatively. Diffuse pericardial thickening with a history of infection (particularly tuberculosis), uraemia, radiation exposure, or prior cardiac surgery would suggest constrictive pericarditis. Haemorrhage may initially appear as an isodense mass and become hypodense as it evolves chronically. While caseous necrosis may be associated with mycobacterial infection, sterile chronic degeneration following Haemorrhage or trauma has also been proposed. In this case, long-standing calcification on prior imaging and negative microbiological and pathological findings supported a benign, non-infectious aetiology. A history of blunt chest trauma has been associated with pericardial cyst formation due to increased intrapericardial pressure by compression between the sternum and vertebrae,^1^^,^^4^ and chronic post-traumatic pericardial haematoma with secondary calcification may represent a plausible mechanism. Previously reported calcified pericardial cyst causing cardiac compression were summarized in **Table S1**.

Surgical resection is indicated for symptomatic patients or when cardiac compression or diagnostic uncertainty exists. Extensive calcification and epicardial adherence favoured a median sternotomy approach in this case. A key surgical principle highlighted by this case is the balance between radical excision and myocardial safety because cysts may become densely adherent to the epicardium or myocardium.^5^ Densely adherent calcified tissue at the atrioventricular groove was intentionally left in situ to avoid RV injury. Intraoperative TEE was critical for confirming adequate decompression despite incomplete calcific removal. Leaving adherent calcified tissue in situ may be prudent to minimize myocardial injury.

Although no standardized follow-up regimen exists, annual TTE surveillance may be reasonable to exclude the progression of residual calcified tissue.

## CONCLUSION

Although rare, giant calcified pericardial cysts with caseous degeneration can cause clinically significant cardiac compression. This case highlights the importance of multimodality imaging and tailored surgical strategy to achieve safe decompression while minimizing myocardial injury.

## Supplementary Material

ivag097_Supplementary_Data

## Data Availability

The data underlying this article will be shared on reasonable request to the corresponding author.
