# Supplementary material for: Giant Calcified Pericardial Cyst With Caseous Degeneration Causing Right Ventricular Compression
Source: Interdiscip Cardiovasc Thorac Surg. 2026 Apr 9;41(5):ivag097. doi: 10.1093/icvts/ivag097 (PMC13187626; doi:10.1093/icvts/ivag097)
Supplement: ivag097_Supplementary_Data [file ivag097_supplementary_data.zip › Supplementary Video Legends.docx]

**Video Legends**

Video 1 Preoperative transthoracic echo image: The right ventricle and tricuspid annulus were compressed by the pericardial cyst.

Video 2 Preoperative Computed Tomography Angiography: Entire inferior wall is covered with calcified pericardial cyst, and right-sided heart was compressed.

Video 3 Intraoperative findings: Calcified cyst wall with caseous material was seen, and the cyst was resected as completely as safely possible.

Video 4 Intraoperative transesophageal echo images before and after cyst resection: Complete relief of right ventricular compression with preserved right ventricular systolic function.
