# Supplementary material for: Giant Calcified Pericardial Cyst With Caseous Degeneration Causing Right Ventricular Compression
Source: Interdiscip Cardiovasc Thorac Surg. 2026 Apr 9;41(5):ivag097. doi: 10.1093/icvts/ivag097 (PMC13187626; doi:10.1093/icvts/ivag097)
Supplement: ivag097_Supplementary_Data [file ivag097_supplementary_data.zip › Supplementary table 1 .docx]

| **Year** | **Author** | **Age, Sex** | **Symptoms** | **Size** | **Location** | **Degree of Calcification** | **Intracystic Content** | **Surgical approach** | **Procedure** |
| --- | --- | --- | --- | --- | --- | --- | --- | --- | --- |
| 1997 | Ng and Olak^S1^ | 66, M | chest dyscomfort | 10 x 6cm | RV outflow tract | circumferencial | necrotic debris | sternotomy | cyst removal |
| 2019 | Sorour^S2^ | 56, M | dyspnea, presyncope | 9.5x8.5x2.0cm | RV wall (cardiophrenic region) | circumferencial | degenerative hemorrhagic material | not specified | pericardiectomy, cyst removal |
| 2021 | Sliman^3^ | 76, M | dyspnea, chest pain, leg edema | 7.5x5.5cm | RV free wall, proximal RCA region | circumferencial | not described | sternotomy | complete pericardiectomy |
| 2022 | Lykins^5^ | 63, M | dyspnea, jaundice | Not described | RV groove | circumferencial | paste-like material, old clot | sternotomy | cyst removal + CABG(SVG-RCA) |
| 2023 | Rahman^2^ | 50, M | dyspnea, cough, syncope | 9x8x4.5cm | Anterior mediastinum to left hemithorax | circumferencial | whitish thick fluid | left thoracotomy | cyst removal |

Supplementary table 1 Case summary of calcified pericardial cyst causing cardiac compression

RV; right ventricle, RCA; right coronary artery, CABG; coronary artery bypass grafting, SVG; saphenous vein graft

[S1] Ng AF, Olak J. Pericardial cyst causing right ventricular outflow tract obstruction. Ann Thorac Surg 1997;63:1147-8

[S2] Soroue AA, Maleszewski JJ, Schaff HV et al. A symptomatic calcified pericardial cyst. Mayo Clin Proc 2019;94:367-9
